# Rotavirus breakthrough infections responsible for gastroenteritis in vaccinated infants who presented with acute diarrhoea at University Teaching Hospitals, Children’s Hospital in 2016, in Lusaka Zambia

**DOI:** 10.1371/journal.pone.0246025

**Published:** 2021-02-04

**Authors:** Julia Simwaka, Mapaseka Seheri, Gina Mulundu, Patrick Kaonga, Jason M. Mwenda, Roma Chilengi, Evans Mpabalwani, Sody Munsaka

**Affiliations:** 1 Department of Pathology and Microbiology, School of Medicine, University of Zambia, Lusaka, Zambia; 2 Department of Biomedical Sciences, School of Health Sciences, University of Zambia, Lusaka, Zambia; 3 World Health Organization Regional Office for Africa (WHO/AFRO), Brazzaville, Congo; 4 Department of Epidemiology and Biostatistics, School of Public Health, University of Zambia, Lusaka, Zambia; 5 Department of Internal Medicine, University Teaching Hospital, Tropical Gastroenterology and Nutrition Group, Lusaka, Zambia; 6 Department of Virology, Diarrhoea Pathogens Research Unit and WHO AFRO Rotavirus Regional Reference Laboratory, South African Medical Research Council, Sefako Makgatho Health Sciences University, Pretoria, South Africa; 7 Center for Infectious Disease Research in Zambia, Lusaka, Zambia; 8 Department of Paediatric and Child Health, School of Medicine, University of Zambia, Lusaka, Zambia; University of Nicolaus Copernicus in Torun, POLAND

## Abstract

**Background:**

In Zambia, before rotavirus vaccine introduction, the virus accounted for about 10 million episodes of diarrhoea, 63 000 hospitalisations and 15 000 deaths in 2015, making diarrhoea the third leading cause of death after pneumonia and malaria. In Zambia, despite the introduction of the vaccine acute diarrhoea due to rotaviruses has continued to affect children aged five years and below. This study aimed to characterise the rotavirus genotypes which were responsible for diarrhoeal infections in vaccinated infants aged 2 to 12 months and to determine the relationship between rotavirus strains and the severity of diarrhoea in 2016.

**Methods:**

Stool samples from infants aged 2 to 12 months who presented to the hospital with acute diarrhoea of three or more episodes in 24 hours were tested for group A rotavirus. All positive specimens that had enough sample were genotyped using reverse transcriptase Polymerase Chain Reaction (RT-PCR). A 20-point Vesikari clinical score between 1–5 was considered as mild, 6–10 as moderate and greater or equal to 11 as severe.

**Results:**

A total of 424 stool specimens were tested of which 153 (36%, 95% CI 31.5% to 40.9%) were positive for VP6 rotavirus antigen. The age-specific rotavirus infections decreased significantly (p = 0.041) from 2–4 months, 32.0% (49/118) followed by a 38.8% (70/181) infection rate in the 5–8 months’ category and subsequently dropped in the infants aged 9–12 months with a positivity rate of 27.2%. 38.5% of infants who received a single dose, 34.5% of those who received a complete dose and 45.2% (19/42) of the unvaccinated tested positive for rotavirus. The predominant rotavirus genotypes included G2P[6] 36%, G1P[8] 32%, mixed infections 19%, G2P[4] 6%, G1P[6] 4% and G9P[6] 3%.

**Discussion and conclusion:**

Results suggest breakthrough infection of heterotypic strains (G2P[6] (36%), homotypic, G1P[8] (32%) and mixed infections (19%) raises concerns about the effects of the vaccination on the rotavirus diversity, considering the selective pressure that rotavirus vaccines could exert on viral populations. This data indicates that the rotavirus vaccine has generally reduced the severity of diarrhoea despite the detection of the virus strains.

## Background

Rotavirus is the primary cause of acute gastroenteritis in children under five years of age worldwide. Rotavirus diarrhoea is attributed to 215,000 deaths worldwide and 121,000 (56%) of the deaths occur in sub-Saharan Africa [1]. Currently, rotaviruses are associated with 24 million diarrhoeal episodes requiring clinic visits, with an estimated 114 million episodes requiring home care per year, and 2.4 million associated hospitalisations globally [2]. Diarrhoea is the third leading cause of death after pneumonia and malaria in Zambia. It caused an estimated 10 million episodes in 2015 which resulted in 63,000 hospitalizations and 15,000 deaths [3]. Because of the high disease burden of rotavirus diarrhoea, the World Health Organisation (WHO) recommended the inclusion of either of the two rotavirus vaccines in the routine immunisation schedule of each country [4]. Zambia introduced Rotarix™, a monovalent, live attenuated vaccine which contains a single human G1P [8] rotavirus strain partially in 2012. The two vaccines have also been tested in developing countries and the efficacy is lower as compared to the efficacy in developed countries [5–7]. The error-prone RNA-dependent RNA polymerase predisposes rotaviruses to genetic mutation and the segmented RNA genome inclines the virus to genome reassortment. These evolutionary mechanisms generate novel strains and have the potential to lead to the emergence of vaccine escape mutants [8]. Reduction and the best prevention measure of severe diarrhoea due to rotaviruses can be attained by using rotavirus vaccines. To fulfil this recommendation, Zambia introduced the live attenuated oral rotavirus vaccine, Rotarix, in its routine immunization schedule in different phases. The first phase was the introduction in four districts (Luangwa, Kafue, Chongwe and Chibombo) of Lusaka province in January 2012 and the follow-up phase was the countrywide roll out in November 2013 [9]. Vaccinated children are still presenting with acute diarrhoea and there is a need to document the genotypes that are causing acute diarrhoea in these vaccinated infants. This prompted the design of this study to determine strains that were contributing to the cause of diarrhoea in vaccinated infants in 2016. Documenting these possible emerging strains that maybe shed after vaccine introduction is important though it is difficult to ascertain the role that the vaccine could have on the emergence and increase of these diverse rotavirus strains, as full genome sequencing was not done to confirm whether they are genetically and structurally different from the one that was used to manufacture the vaccine.

## Methods

In this study, ethical approval was granted by the University of Zambia Biomedical Research Ethical Committee (UNZABREC) after which a waiver and permission to conduct the study was given (28^th^ September, 2017) reference number 067-06-17. Since this study used stool samples which were collected for surveillance purposes in 2016 and there was no direct contact with participants; no informed consent was obtained from parents/guardians of the participants.

Stool samples were selected retrospectively from a pool that was collected for sentinel surveillance for acute gastroenteritis at the University Teaching Hospitals (UTHs), Children`s Hospital in Lusaka, Zambia. Systematic sampling targeted samples from infants aged 2 to 12 months who presented to the hospital with acute diarrhoea of three or more episodes in 24 hours. The stool samples were tested for group A, targeting Viral Protein (VP) 6 antigen using an ELISA kit (ProSpecT^TM^, Oxford, UK). All positive specimens that had enough sample were genotyped using reverse transcriptase Polymerase Chain Reaction (RT-PCR). A 20-point Vesikari clinical score was used to determine the severity of acute diarrhoea in infants under study. Diarrhoea scores between 1–5 were considered as mild, 6–10 as moderate and between 11–20 as severe.

Out of 823 under five years’ cases that were enrolled for acute diarrhoea surveillance at UTHs Children`s hospital in 2016, 512 stool specimens were systematically selected, without replacement, for this study. Out of the 512 stool specimens, 424 (83.0%) met the case definition of acute diarrhoea (infants having ≥3 or more loose stool episodes in 24 hours) were included in the study. A total of 88 cases were excluded from the study. The 424 stool samples selected were tested for group A human rotavirus targeting the VP6 antigen, using enzyme immunoassay (EIA, ProSpect^TM^, Oxoid, UK), following the manufacturer`s instructions.

Positive stool samples that had enough sample (116 samples, 75.8%) were stored at -20°C before being genotyped at Sefako Makgatho Health Sciences University, South African Medical Research Council, Diarrhoeal Pathogens Research Unit and Word Health Organisation (WHO) AFRO Rotavirus Regional Reference Laboratory, Department of Virology, Pretoria, South Africa, to characterise the rotavirus genotypes. Rotavirus dsRNA extraction was performed from 140 ml of a 10% faecal suspension prepared by diluting a pea-size stool specimen or 100μl of watery diarrhoea in distilled water. A QIAamp viral RNA mini kit (Qiagen, Hilden, Germany) was used to extract dsRNA per the manufacturer’s instructions. We performed RT-PCR according to previously described protocols complied in the WHO manual of rotavirus detection and characterisation methods [4]. The primer pair sBeg/End9 was used to reverse-transcribe the full-length VP7 gene (1062 bp) [10]. While Con2/Con3 primer set was used to amplify a partial-length of VP4 gene (876 bp) as described by Gentsch et al. [11]. The rotavirus genotyping assays for G-types were carried out using a semi-nested multiplex PCR with type-specific primers (RVG or EndA and aAT8v, aBT1, aCT2, aDT4,mG3, mG9, mG10 and G12) to determine G1, G2, G3, G4, G8, G9, G10 and G12 genotypes [10, 12, 13]. Type-specific primers (dP[8], 2 T-1, 3 T-1, 4 T-1, 5 T-1, P[11] and P[14]) with Con3 were used to determine P[4], P[6], P[8], P[9], P[10], P[11] and P[14] genotypes [11, 12, 14]. Amplification cycling conditions used were as follows; 30 cycles of denaturation at 94°C for 1 minute, annealing at 42°C for 1 minute, extension at 72°C for 1 minute, with the final extension at 72°C for 7 minutes [15]. The reaction was held at 4°C until the reaction tubes were removed and electrophoresis was done to visualize the amplicons. A 100 bp molecular weight marker was used to determine the size of the amplicons and classify them accordingly. Negative and positive controls were included in all the reactions for both VP7 and VP4 genes. A type-specific nested PCR assay was used to confirm genotypes in mixed infection samples.

Data were entered using Epi data version 3.1(120306), (Epidata Association, Jens M. Laurisen Michael Bruus, Denmark) and exported to Stata version 13.1 (Stata Corp, College Station, Texas, USA) for final analysis. The comparison of frequencies between positive and negative rotavirus groups was done using proportions. Continuous data were tested for normality using the Shapiro-Wilk test in Stata and was found to be skewed. A 20-point Vesikari clinical score was used to determine the severity of acute diarrhoea in infants under study. Parameters for clinical assessment of diarrhoea severity included: duration and the maximum number of episodes of diarrhoea and vomiting, the intensity of fever and dehydration. As per the Vesikari score, [16] a grade of 1–5 was considered as mild, 6–10 as moderate and 11–20 as severe.

The significance was set at a P-value of less than 0.05.

## Results

The demographic and clinical characteristics of the infants aged 2–12 months who were hospitalized for acute gastroenteritis according to their rotavirus status is displayed in Table 1. Out of 424 stool samples that were tested, 245 (57.8%) were male and 179 (42.2%) were female infants. Out of the 245 males tested for rotavirus diarrhoea, 96 (39.2%) had acute rotavirus diarrhoea, and out of 179 female infants tested, 57 (31.8%) were infected with rotavirus. Both male and female infants had the same chance of having acute rotavirus diarrhoea (p = 0.126).

**Table 1:** Demographic and clinical characteristics of infants who presented with acute diarrhoea at University Teaching Hospitals–Childrens’ Hospital.

Characteristic	Total = 424 N (%)	Positive Rotavirus N (%)	Negative Rotavirus N (%)	P-value
Sex				
Male	245 (57.8)	96 (39.2)	149 (60.8)	0.126
Female	179 (42.2)	57 (31.8)	122 (68.2)	
Age category (months)				
2–4	118 (27.8)	49 (41.5)	69 (58.5)	**0.041**
5–8	181 (42.7)	70 (38.8)	111(61.3)	
9–12	125 (29.5)	34 (27.2)	91 (72.8)	
Treatment				
ORS	122 (28.8)	35 (28.7)	87(71.3)	0.123
IVF	238 (56.1)	94 (39.5)	144 (60.5)	
ORS + IVF	64 (15.1)	24 (37.5)	40 (62.5)	
Rotavirus vaccine received				
1 dose	52 (12.3)	20(38.5)	32(61.5)	0.351
2 doses	330(77.8)	114(34.5)	216(65.5)	
Unvaccinated	42(9.9)	19(45.2)	23(54.8)	
Duration of diarrhoea (days)				
1–4	385 (90.8)	141 (36.)	244 (63.4)	0.402
5–6	27 (6.4)	10 (37.0)	17 (63.0)	
≥7	12 (2.8)	2 (16.8)	10 (83.3)	
Diarrhoea episodes (24hrs)				
3–5	307 (72.4)	110 (35.8)	197 (64.2)	0.969
6–10	112 (26.4)	41 (36.6)	71 (63.4)	
≥ 11	5 (1.2)	2 (40.0)	3 (60.0)	
Duration of vomiting (days)				
No vomiting	185 (43.60	70 (37.8)	115 (62.2)	0.188
1	60 (14.2)	16 (26.7)	44 (73.3)	
2	89 (21.0)	38 (42.7)	51 (57.3)	
3	90 (21.2)	29 (32.2)	61 (67.8)	
Vomiting episodes (24hrs)				
No vomiting	185 (43.6)	70 (37.8)	115 (62.2)	0.219
1 emesis	43 (10.1)	18 (41.9)	25 (58.1)	
2–4 emesis	116 (27.4)	33 (28.5)	83 (71.5)	
≥ 5 emesis	80 (18.9)	32 (40.0)	48 (60.0)	
Temperature (°C)				
37–38.4	306 (72.2)	101 (33.0)	205 (67.0)	**0.010**
38.5–38.9	47 (11.1)	15 (31.9)	32 (68.1)	
39–41.2	71 (16.7)	37 (52.1)	34 (47.9)	

ORS = oral rehydration salts; IVF = intravenous fluid.

Overall, there was a significant age-specific association between infants who had rotavirus diarrhoea and those that were negative for rotavirus (p = 0.041). The age-specific rotavirus infections increased from 2–4 months, 49/118 (41.5%), peaked in the 5–8 months’ category 70/181 (38.8%) and subsequently dropped in the infants aged 9–12 months 34/125 (27.2%). Infants who had acute rotavirus diarrhoea did not differ significantly from those who did not have rotavirus diarrhoea in the type of treatment that they received (p = 0.123), even though most infants who presented to the hospital with acute diarrhoea received intravenous fluid 238/424 (56.1%).

Infants who had rotavirus acute diarrhoea were not significantly different from those that did not have rotavirus diarrhoea in the number of episodes of diarrhoea (p = 0.969), episodes of vomiting (p = 0.219), duration of diarrhoea and vomiting (p = 0.402. and 0.188 respectively). Infants who received 1 dose or 2 doses and those not vaccinated at all had the same chance of having acute diarrhoea due to rotavirus (p = 0.351). Infants who had acute rotavirus gastroenteritis differed significantly from those who were negative for rotavirus by fever (p = 0.010). The median duration of acute diarrhoea in days was 3 days (IQR, 2–3), and the median episodes of diarrhoea in 24 hours were 5 episodes (IQR, 4–6). The median duration of vomiting was 2 days (IQR, 1–3) while the median episode of vomiting in 24 hours was 3 (IQR, 2–4).

**Fig 1:**
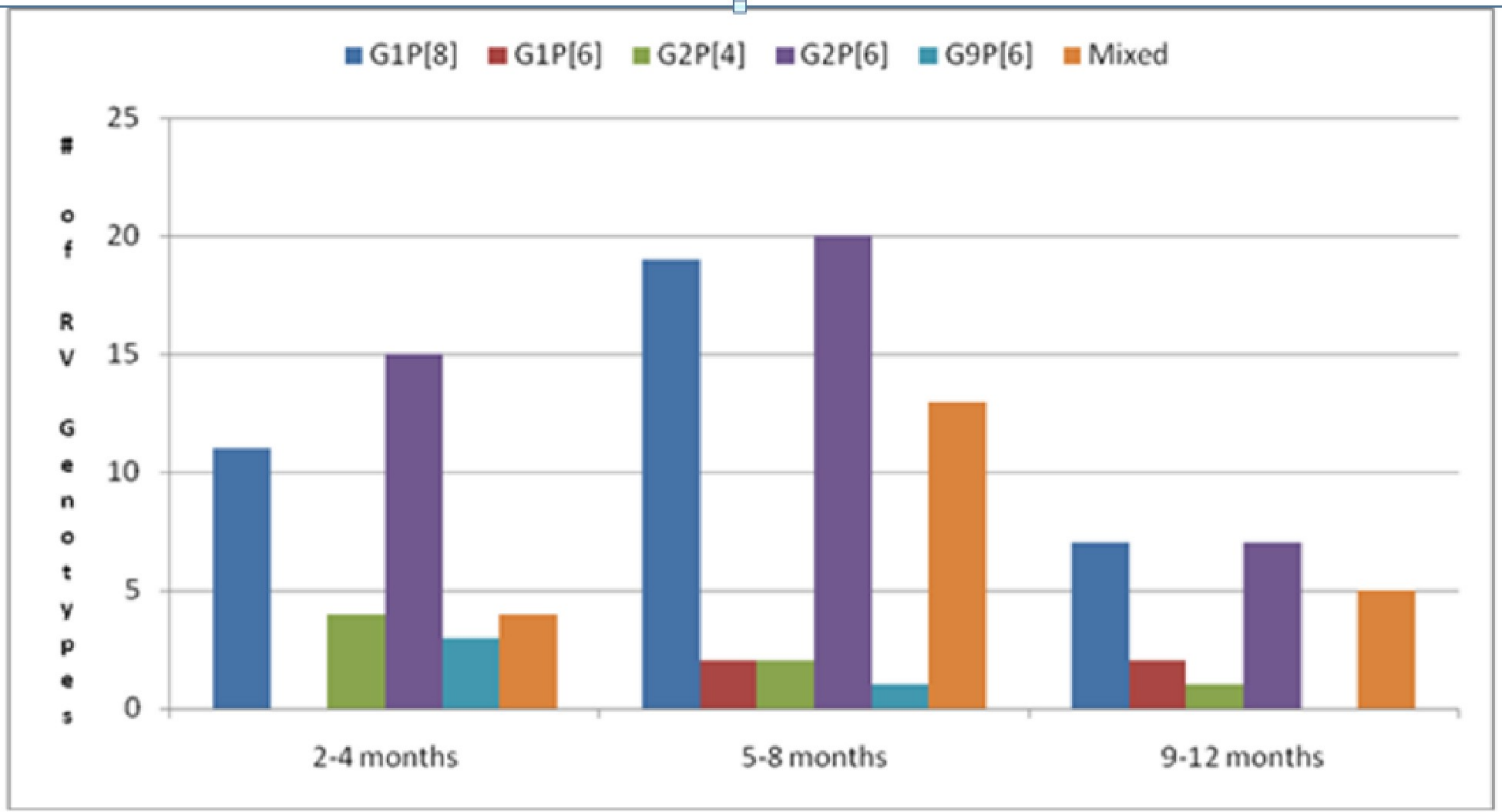
Age distribution of group A rotavirus genotypes which infected infants who presented with acute diarrhoea at the UTHs Children’s Hospital in 2016.

Overall, there was no significant difference in the genotypes that infected infants aged 2–12 months who presented with acute diarrhoea in 2016 (Fig 1). Most infants aged 2–4 months had acute diarrhoea due to G2P[6] 15/42 (35.7%) followed by G1P[8] 11/37 (29.7%). Only 4/22 (18.2%) of rotavirus infections that occurred in this age group were due to mixed genotype infections. A few infections were caused by G2P[4] 4/7 and G9P[6] 3/4. There were no G1P[6] infections in this age group. Infants age 5–8 months were infected with all rotavirus genotypes which were detected in this study with most infections being caused by G2P[6] 20/42 (47.6%), followed by G1P[8] 19/37 (51.4%) and mixed infections 13/22 (59.1%). The older age group, 9–12 months were infected with G1P[8] 7/37 (18.9%), G2P[6] 7/42 (16.7%) and mixed infections 5/22 (22.7%).

**Fig 2:**
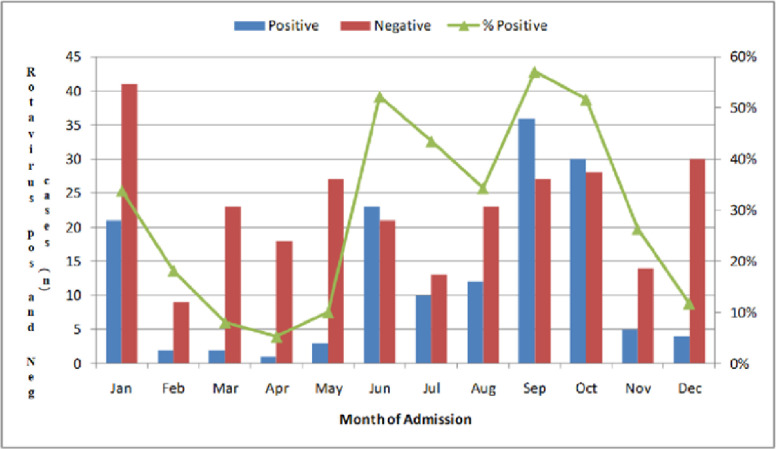
Monthly distribution rotavirus infections detected in infants who presented with acute diarrhoea at UTHs Children’s Hospital in 2016.

Out of 424 samples which were eventually tested for human group A rotavirus VP6 antigen, 153 stool samples, representing 36.1%, (95% CI 31.5% to 40.9%) were positive for rotavirus VP6 antigen and 271 (64%) were negative. Rotavirus acute diarrhoea occurred throughout the year with the highest pick occurring in the cool dry months through to the dry hot months of the year (Fig 2).

**Fig 3:**
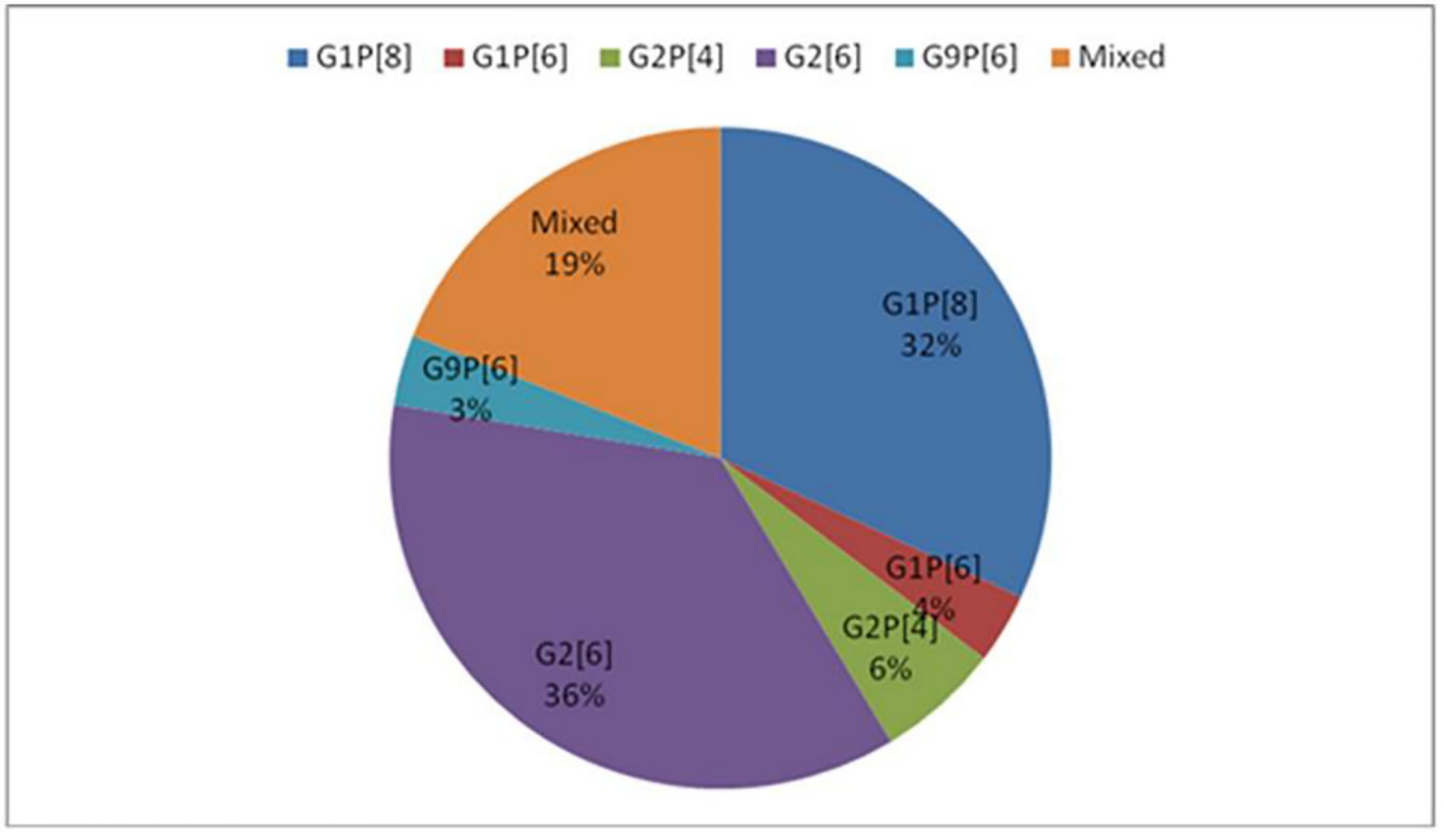
Rotavirus genotypes which were responsible for the acute diarrhoea in infants aged 2–12 months at UTHs Children’s Hospital in 2016.

**Fig 4:**
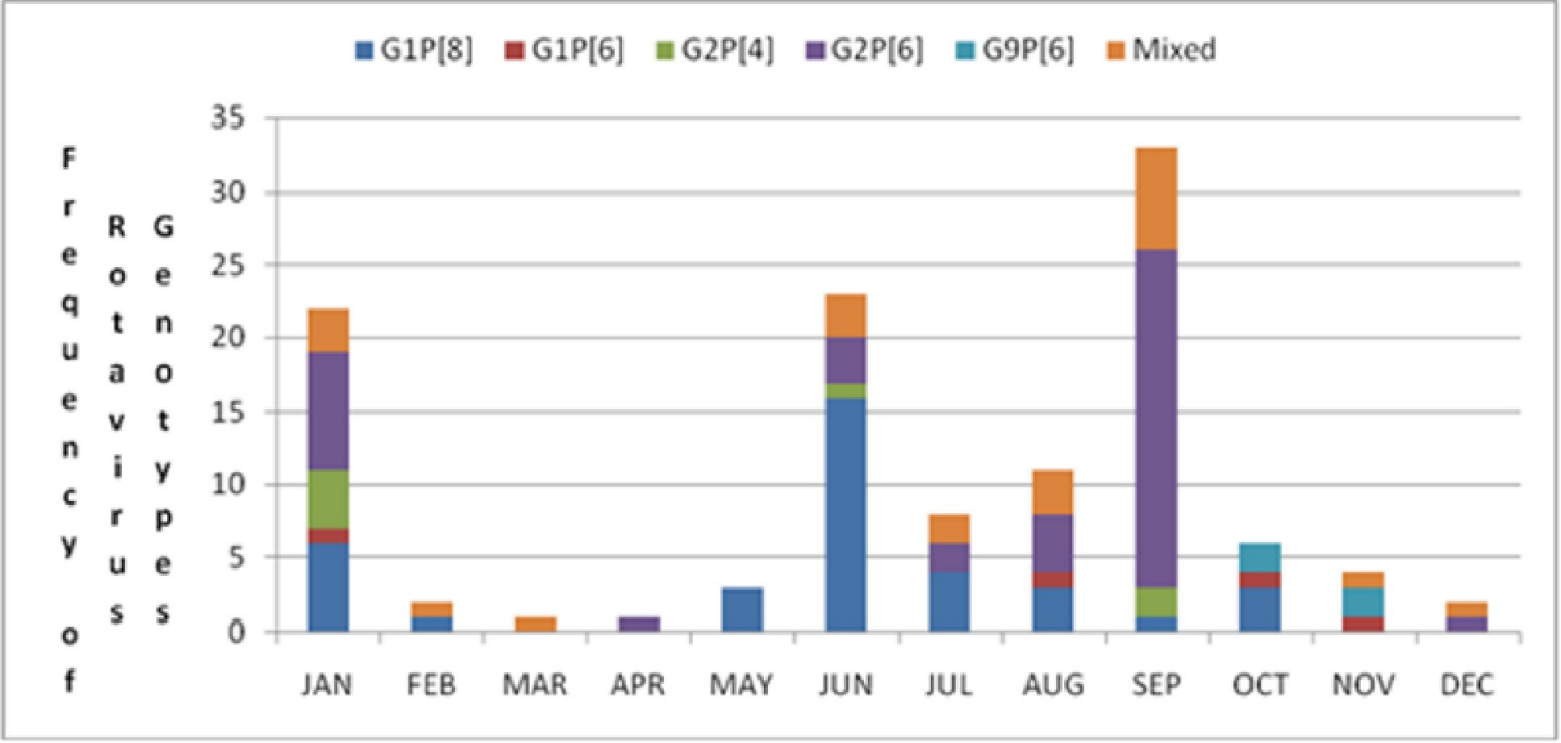
Monthly variation of acute diarrhoea infections which occurred at UTHs Children’s Hospital in 2016 due to different rotavirus strains.

Most rotavirus infections which occurred in infants in this study were because of G2P[6] 42/116 (36%), followed by G1P[8] 37/116 (32%), mixed infections 22/116 (19%). Rotavirus genotypes that were responsible for the dual infections that occurred in the infants who were studied included, G9P[6]/P[8], G2P[6]/P[4], G1P[8]/P[4]/P[6], G1/G2P[6], G1P[4]/P[8], G-P[8]/P[4], G8/G3P[6], G3/G9P[6], G1P[6]/P[8], G1/G2P[4], G2/G9 P[6]/P[8]G2 P[6]/P[4] G2/G9 P[6], G9P[6]/P[8], G1/G9P[6], G1/G12P[6]. G2P[4] was only responsible for 7/116 (6%), G1P[6] 4/116 (4%.) and a few G9P[6] 4/116 (3%) as shown (Fig 3). Fig 4 shows that monthly variation of acute diarrhoea infections which occurred in infants aged 2–12 months at the University Teaching Hospitals Children’s Hospital in 2016. Many infections which occurred in January 2016 were as a result of G2P [6] 8/22 (36.4%); followed by G1P[8] 6/22 (27.3%); followed by G2P[4] 4/22 (18.2%) and a few mixed infections 3/22 (18.2%). There was a reduction in acute diarrhoea cases due to rotavirus in February through to May 2016, with a few infections that occurred in these months being caused by G1P[8], mixed infections and G2P[6]. Most acute diarrhoea infections which occurred in June (the coolest month in Zambia) were as a result of G1P[8] 16/23 (69.6%), followed by G2P[6] 3/23(13.0%), mixed infections 3/23 (13.0%) and one infection was due to G2P[4] 1/23 (4.4%).

The greater part of infections that occurred in July was due to G1P[8] 4/8 (50.0%) followed by G2P[6] 2/8 (25.0%) and mixed infections 2/8 (25.0%). In August, 11 acute diarrhoea infections which occurred in this month were because of G2P[6] 4/11 (36.4%) followed by G1P[8] 3/11 (27.3%), mixed infections 3/11 (27.3%) and one infection which was caused by G1P[6] strain. September is the beginning of the hot dry season in Zambia and that is where most rotavirus infections 33/116 took place. The common genotype which caused acute diarrhoea in infants who presented to the hospital in this month was G2P[6] 23/33 (69.7%). The rest of the acute rotavirus diarrhoea which occurred in September was due to mixed infections 7/33 (21.2%), G2P[4 2/33 (6.1%) and G1P[8] 1/33 (3.0%). There was a reduction of acute diarrhoea due to rotavirus in October through to December in 2016, with a few rotavirus acute diarrhoea infections being caused by G1P[8], G1P[6], G9P[6], G2P[6] and mixed infections.

**Fig 5:**
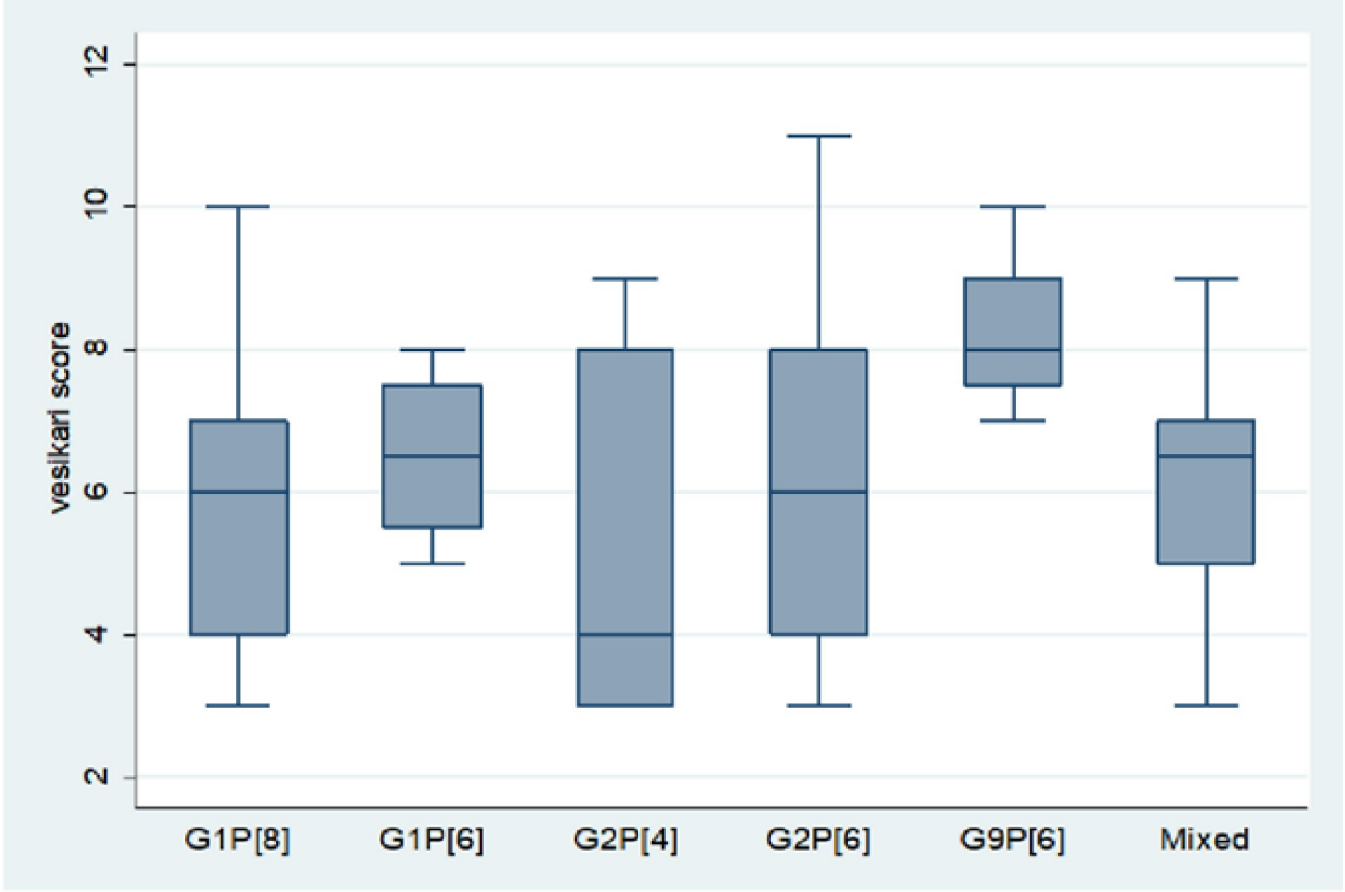
The relationship between the rotavirus strains that caused acute diarrhoea and its severity.

Most rotavirus acute diarrhoea infections which occurred in 2016 were mild to moderate (Fig 5). The median Vesikari score for acute diarrhoeal infections the occurred due to G1P[8] and G2P[6] was 6, indicating moderate infections with Vesikari score between 6–10. The median Vesikari score for infections which were due to G1P[6] and mixed infections was 7, causing moderate acute diarrhoea with Vesikari scores ranging between 6–10, while 4 and 8 were the median Vesikari scores for infections caused by G2P[4] and G9P[6] respectively. Acute diarrhoea infections which occurred in 2016 were mild to moderate infections; we did not record any severe diarrhoea except for 3 cases which were due to G2P[6] and had a Vesikari score of 11.

## Discussion

This study demonstrates that slightly over a third (36.1%) of infants aged 2–12 months who were admitted at the UTHs Children’s Hospital due to acute diarrhoea had rotavirus infection. This prevalence is slightly higher than the one reported in another study carried out in Lusaka by Mpabalwani and colleagues, who found the prevalence of rotavirus acute diarrhoea to be 24.7% in 2014 and 27% in 2015 [17]. This observation may be due to the difference in the study participants and the period of analysis. In the previous study, all children below the age of five were analysed for over 2 years, while this study looked at infants aged 2–12 months for 12 months. A study conducted in Rwanda documented a decline in the proportion of hospitalisations due to rotavirus diarrhoea among children <1 year of age [18]. Another study conducted in Zimbabwe reported a rotavirus disease burden of 25% in children below the age of five after the vaccine was introduced [19]. A study conducted in Kenya observed a prevalence of rotavirus diarrhoea of 14.5% after the vaccine was introduced in their routine immunisation schedule [20]. These variations may reflect actual differences in rotavirus gastroenteritis disease burden, but may also be related to variations in study design, the methods used in detecting rotavirus, and vaccination against rotavirus diarrhoea.

This study demonstrates that there is an age-specific significant difference in the burden of rotavirus infection in infants who were studied (p = 0.041). These findings are consistent with the study that was conducted in Nepal where it was found that the highest prevalence of rotavirus diarrhoea occurred in infants aged 0–11 months (26.7%) followed by 23.6% in the 12–23 months [21].

Rotavirus infections in 2016 occurred throughout the year, with the highest peak being in June, 52% (cool dry month), September and October (hot dry months) 57% and 52% respectively. These findings are consistent with the findings documented in a study conducted in Nigeria where they had a marked seasonal peak, with more than 90% of rotavirus infections occurring during December to March, their cool dry months [22]. Similar results were documented from a study conducted by Mwenda and colleagues who showed that rotavirus infections occurred throughout the year in other African countries (Uganda, Ghana, and Ethiopia), with a peak prevalence generally being recorded during the cool dry months of the year [23].

A diversity of rotavirus genotypes, G2P[6] 36%, G1P[8] 32%, mixed infections 19%, G2P[4] 6%, G1P[6] 4%, and G9P[6] 3% were predominant in 2016. These findings are consistent with the findings of Seheri and colleagues who found that the predominant genotypes circulating in sub-Saharan Africa included G1P[8] (23.8%), followed by G2P[4] (11.8%), G9P[8] (10.4%), G12P[8] (4.9%), G2P[6] (4.2%) and G3P[6] (3.7%) [24]. The findings are also consistent with the report of the African Rotavirus Surveillance Network among hospitalised children aged <5 years which showed a high diversity of circulating rotavirus strains in the subcontinent [23]. According to this study findings, G2P[6], G1P[8] and mixed infections caused most of the acute diarrhoea infections which occurred in 2016. These breakthrough infections reached their highest peaks in January, June and September with G2P[6] being predominant in January and September and G1P[8] in June. Rotarix, a monovalent rotavirus vaccine is expected to provide cross-protection against the circulating rotavirus genotypes that share either the G or the P-type with the vaccine strain G1P[8] (homotypic protection) [25]. In this study, 77.8% of infants who presented with rotavirus diarrhoea received a complete dose (2 doses), 12.3% received incomplete (1 dose) and 9.9% were not vaccinated. It is important to note that most rotavirus infections which occurred in fully vaccinated infants were caused by G2P[6] (38.4%), followed by G1P[8] 27.9% and mixed infections 19.8%. There is a need to do a full genome analysis of G1P[8] to establish whether infection being caused by this genotype are wild or vaccine-derived. It is also cardinal to continuously monitor the presence of G2P[6] to determine whether the increase in this genotype which is seemingly evading the vaccine immunity is due to vaccine pressure or genotype evolution. Vaccine trial studies conducted in Malawi and South Africa in 2012 indicated that significant protection was afforded by the vaccine against severe gastroenteritis caused by G2P[4], a heterotypic rotavirus strain (vaccine efficacy against G2: 79.2% [95% CI: 8.9%; 96.5%; p 0.017]; vaccine efficacy against P[4]: 70.9% [95% CI: 37.5%; 87.0%]) [25]. There is need to do a strain-specific vaccine effectiveness study to confirm the efficacy of the Rotarix vaccine against P[6] since there is limited data concerning the vaccine efficacy of P[6] vaccine in Africa.

The G2P[6], a fully heterotypic genotype to the Rotarix vaccine, infections started increasing after the vaccine was introduced in Lusaka in 2012, and became predominant throughout the post-vaccine period as indicated in another study [26]. These changes in circulating genotypes after vaccine introduction are consistent with what has been observed in other countries [27–32].

G2P[6] strains have a different overall genomic RNA constellation and hence belong to a different genogroup (DS-1) than the Rotarix vaccine strain which belongs to Wa-like genogroup [33]. Since Rotarix is a single live attenuated monovalent human G1P[8] strain vaccine, the continued breakthrough infections caused by G1P[8] genotype also raises concerns and suggest further investigations by doing a full genome analysis to rule out vaccine-derived acute diarrhoea in infants who are vaccinated with Rotarix vaccine

Other studies have linked rotavirus susceptibilities to Histo-Blood Group Antigens (HBGA) phenotype of the host [34, 35]. Depending on the P genotype (either P [4], P [6] and P [8]), HBGA binding is different. Non- secretor individuals have an active α 1,2 fucosyltransferase and they do not synthesize the H-type 1 antigen thus they are not susceptible to P[6] rotavirus genotype infections. P [4] and P [8] bind specifically by recognizing both the common Le^b^ as the H-type 1 antigen while P[6] genotypes completely recognize the H-type 1 [36]. The observation made by Heylen and colleagues suggested that gene segment 4 is the main differentiator between human P[6] and non-P[6] strains suggesting that the VP4 spike protein is most likely one of the main reasons preventing the rapid spread of P[6] strains to the rest of the world despite multiple introductions [37]. These findings from other studies suggest further investigation of Zambia’s children below the age of five to investigate the type of secretor status that is common given the substantial amount of G2 P [6] infections in the present study. The finding would also highlight how much of low vaccine efficacy observed in Africa is attributable to the secretor status phenomenon.

These findings are slightly different with what was found in Nicaragua, where it was found that G12 genotype was responsible for most of the diarrhoea hospitalisation that they recorded in children below the age of five after the vaccine was introduced [38]. In Colombia, a study conducted by Pelaez-Carvajal and colleagues demonstrated that G2P [4], a strain fully heterotypic to the Rotarix vaccine, was predominant before vaccine introduction in 2008 (47%) and after vaccine introduction in 2010, G2P [4] was responsible for 54% of diarrhoeal infections observed in infants in the same year, 86% in 2011 and 32% in 2012 [39] Pelaez-Carvajal and colleagues suggested that surveillance data should be mixed with information on disease incidence and strain-specific vaccine effectiveness to avoid misinterpretation about the role of vaccine pressure on the emergence of new or persistent strains [39]. Another study conducted by Paulke-Korinek and colleagues demonstrated that G2P [4] genotype caused most of the breakthrough infection in vaccinated children in Austria [40]

The presence of G2P [6], before the introduction of the vaccine, although at a lower rate (5%) [26], and its prevalence after the vaccine was introduced may suggest secular variation rather than vaccine pressure as a cause for the fluctuation. The breakthrough infection of heterotypic strains (G2P[6] (36%), homotypic, G1P[8] (32%) and mixed infections (19%) raises concerns about the effects of the vaccination on the rotavirus diversity, considering the selective pressure that rotavirus vaccines could exert on viral populations. There is need to do a full genome analysis of G1P[8] to rule out the possibility of vaccine-derived G1P[8] infections, considering that the Rotarix vaccine being used in the routine immunisation schedule is a G1P[8] attenuated monovalent vaccine.

## Conclusion

The study provides information on the breakthrough rotavirus genotypes which contributed to diarrhoeal infection in vaccinated infants in 2016 in Lusaka, Zambia. These genotypes included (G2P[6] (36%), G1P[8] (32%) and mixed genotype infections (19%). Overall, there was no significant difference in the genotypes that infected infants aged 2–12 months who presented with acute diarrhoea in 2016. These results. These results may suggest a cyclic variation of detected genotypes rather than vaccine pressure as a cause for the fluctuation, even though such a conclusion would be confirmed by doing a full genome analysis of the circulating genotypes.
